# Longitudinal changes in somatic symptoms and family disagreements among depression and community groups: a 23-year study

**DOI:** 10.1186/s12888-015-0619-2

**Published:** 2015-10-08

**Authors:** Xiaoyu Bi, Jessica Y. Breland, Rudolf H. Moos, Ruth C. Cronkite

**Affiliations:** Center for Innovation to Implementation, Health Services Research and Development (HSR&D), Veterans Affairs Palo Alto Health Care System (152-MPD), 795 Willow Road, Menlo Park, CA 94025 USA; Department of Psychiatry and Behavioral Sciences, Stanford University School of Medicine, 401 Quarry Rd, Stanford, CA 94304 USA; Department of Sociology, Stanford University, 450 Serra Mall, Stanford, CA 94305 USA; Center for Primary Care and Outcomes Research, Stanford University, 117 Encina Commons, Stanford, CA 94305 USA

**Keywords:** Somatic Symptoms, Family disagreements, Depression, Longitudinal, Latent growth curve modeling

## Abstract

**Background:**

Few longitudinal studies describe the relationship between somatic symptoms and family disagreements. We examined changes over time in somatic symptoms, family disagreements, their interrelationships, and whether these patterns differed between individuals treated for depression (depression group) and individuals from the same community (community group).

**Methods:**

We followed participants in the depression (*N* = 423) and community (*N* = 424) groups for 23 years (the community group was matched to the depression group on socioeconomic status, gender, and marital status). All participants were age 18+ and completed surveys at baseline, 1, 4, 10, and 23 year follow-ups. We assessed somatic symptoms and family disagreements at each time point and used latent growth curve modeling to examine change in these constructs over time.

**Results:**

Somatic symptoms and family disagreements changed differently over time. Somatic symptoms decreased between baseline and the 10 year follow-up, but increased between the 10 and 23 year follow-ups, whereas family disagreements decreased linearly over time. Somatic symptoms and family disagreements were higher at baseline and declined at a faster rate in the depression compared to the community group. The relationship between changes in somatic symptoms and changes in family disagreements was also stronger in the depression group: a larger decrease in somatic symptoms was associated with a larger decrease in family disagreements.

**Conclusions:**

Longitudinal changes in somatic symptoms and family disagreements differ between depression and community groups. Individuals treated for depression had more somatic symptoms and family disagreements at baseline and improved at a faster rate compared to individuals in the community. Somatic symptoms and family disagreements may be important targets when treating depression, given the strong interrelationship among these factors in individuals with depression.

## Background

Perceptions of health and family functioning are important components of quality of life [1, 2]. They are also dynamic constructs that may change over time as individuals age and domains of family disagreements evolve (e.g., marital or parent-child disagreements). However, few studies describe longitudinal changes in somatic symptoms, family disagreements, or the association between changes in somatic symptoms and changes in family disagreements. Further, few studies examine how these longitudinal changes and associations differ by mental health status, for example, among individuals treated for depression (i.e., a depression group) and a community sample (i.e., a community group).

### Changes in somatic symptoms and family disagreements over time

The results of longitudinal studies assessing changes in somatic symptoms are mixed. Studies with short follow-up periods (two years or less) find that self-reported somatic symptoms decrease over time among depression and community groups [3–5]. However, findings from studies with longer follow-up periods suggest that somatic symptoms have a non-linear trajectory [6, 7]. For example, Sutin and colleagues found that, among a community sample, somatic symptoms decreased from early adulthood to middle adulthood, and then increased in older adulthood [6, 7].

The data on changes in family disagreements over time are less clear and results from these studies may not be generalizable because most assess only one domain of family disagreements (usually marital conflict). In addition, these studies have mixed results. For example, studies of marital conflict with short follow-up periods suggest that marital conflict is stable over time [8], whereas studies with longer follow-up periods (e.g., 8–20 years) suggest that it increases over time [9, 10], or has a variable course over time (e.g., an essentially stable period followed by a slight decline [11]). There is even less information on how multiple domains of family disagreements change over time. To our knowledge, only one longitudinal study assessed multiple domains of family disagreements over time, suggesting that family disagreements decrease over time [12, 13].

Most data on somatic symptoms and family disagreements are cross-sectional and thus do not allow for an examination of the interrelationships between somatic symptoms and family disagreements over time. For example, using a cross-sectional sample, Dorner and colleagues found a positive association between multiple domains of “family discomfort” and somatic symptoms among adults [14]. The limited longitudinal research on this topic suggests a positive association between family disagreements (in this case marital) and somatic symptoms – i.e., in comparison to women without marital distress, women with marital distress initially had more severe somatic symptoms and a greater decrease in the number of somatic symptoms over 5 years [6].

### The role of depression

Depression is likely an important factor in the association between somatic symptoms and family disagreements over time because longitudinal research suggests that depression affects changes in somatic symptoms, changes in family disagreements, and the association between changes in somatic symptoms and family disagreements [6, 12, 13, 15–18]. For example, the association between somatic symptoms and family disagreements was stronger among participants with depression than among participants without depression over a short follow-up period [15]. Another study among women experiencing martial distress found that, over a 5 year period, women with depression reported an increase in physical symptoms whereas women without depression reported a decrease in physical symptoms [6].

### Present study

The primary purpose of the present study was to examine changes over time in somatic symptoms, family disagreements, and their interrelationships, taking into consideration the limitations of prior work. The present study assessed somatic symptoms and family disagreements at 5 time points over 23 years, assessed multiple domains of family disagreements, and focused on whether changes in somatic symptoms and family disagreements differed among individuals treated for depression and a matched community sample (referred to as depression and community groups, respectively).

The study addressed three research questions: (1) How do somatic symptoms change over time and do changes differ between depression and community groups? (2) How do family disagreements change over time and do changes differ between depression and community groups? (3) Are changes in somatic symptoms associated with changes in family disagreements over time and does the association differ between depression and community groups?

Based on prior studies, we had the following hypotheses. (1) Somatic symptoms would show a non-linear pattern of change over time with an initial decrease in somatic symptoms followed by an increase in somatic symptoms as the cohort aged. We also hypothesized that the depression group would have a higher baseline number of somatic symptoms in comparison to the community group. (2) Family disagreements would be relatively stable or slightly decrease over time, with a larger baseline number in the depression group. (3) Somatic symptoms and family disagreements would be positively associated over time, with stronger associations in the depression group.

## Methods

### Sample and procedure

The depression group (*N* = 423) was comprised of individuals, aged 18+, who received treatment for unipolar depression at one of five facilities in Northern California and met criteria for depression as assessed by the Research Diagnostic Criteria [19]. Participants with diagnoses of a neuropsychological, metabolic, manic, or substance use disorder were excluded. The community group (*N* = 424) was matched to the depression group on socioeconomic status by randomly selecting community participants from each depressed participant’s census tract. Community participants were then matched on gender and marital status. Participants in both groups completed surveys at 5 time points: baseline (T1), 1 year (T2), 4 years (T3), 10 years (T4), and 23 years (T5) follow-up. Participants signed consent forms at each time point. Overall, 90 % of participants completed the survey via mail and 10 % completed the survey over the phone or in person. The study was approved by the Stanford University Institutional Review Board.

Response rates decreased over time due to mortality and attrition. For the depression group, response rates among living participants were 95 % (*N* = 395), 91 % (*N* = 370), 84 % (*N* = 313), and 79 % (*N* = 248) at T2, T3, T4, and T5. For the community group, response rates among living participants were 96 % (*N* = 405), 93 % (*N* = 387), 84 % (*N* = 333), and 79 % (*N* = 272) at T2, T3, T4, and T5. There were few differences in somatic symptoms and family disagreements among participants who did and did not participate in the study in later waves. However, compared to participants who provided data at T3 and T4, participants who provided data at T3, but not T4 had more somatic symptoms and more family disagreements. The present study used the full baseline sample for both the depression group (excluding one participant missing data on all outcome variables) and the community group.

### Measures

All data were self-report and were obtained using the Health and Daily Living Form [20]. Outcome variables (i.e., somatic symptoms and family disagreements) were collected at all 5 time points. Covariates (age, gender, race/ethnicity, education, and medical conditions) were assessed at baseline.

#### Somatic symptoms

Participants reported whether they had experienced any of the following physical symptoms over the past 12 months (0 = no, 1 = yes): (1) acid stomach or indigestion, (2) suddenly felt hot all over, (3) heart beating, hard, pounding, (4) poor appetite, (5) nervousness (fidgety, tense), (6) restlessness, couldn’t sit still, (7) felt weak all over, (8) cold sweats, (9) hands trembling, (10) headaches, (11) constipation, and (12) insomnia (trouble falling or staying asleep). The total number of somatic symptoms ranged from 0 to 12, with higher scores indicating a greater number of somatic symptoms. Internal reliability was good: Cronbach’s alpha ranged from .78 to .82 for the depression group and .75 to .82 in the community group over the course of the study.

#### Family disagreements

Participants reported family disagreements in 14 domains (0 = no, 1 = yes), including disagreements about friends, relatives, driving habits, politics, money, use of the car, watching TV, helping with household chores, sex, drugs, alcohol, cigarette smoking, discipline, and major purchases. The total number of domains of family disagreements ranged from 0 to 14, with higher scores indicating a greater number of domains of family disagreements. Internal reliability was good: Cronbach’s alpha ranged from .74 to .78 in the depression group and .69 to .75 in the community group over the course of the study.

#### Covariates

Older adults, women, non-White individuals, individuals with less education, and individuals with more medical conditions tend to report more somatic symptoms and family disagreements [7, 9–11, 21–24]. Therefore, age, gender (0 = male, 1 = female), race/ethnicity (0 = White, 1 = non-White), number of years of education, and number of diagnosed medical conditions were assessed as covariates. To assess diagnosed medical conditions, participants reported whether they had any of the following conditions in the past 12 months (0 = no, 1 = yes): anemia, asthma, arthritis or rheumatism, bronchitis, cancer, chronic liver trouble, diabetes, serious back trouble, heart trouble, high blood pressure, kidney trouble, stroke, tuberculosis, or ulcer. The total number of diagnosed medical conditions ranged from 0 to 14.

### Analyses

We used latent growth curve modeling (LGM) to address all three research questions. Analyses generally included three steps. First, we fit separate (univariate) unconditional models (i.e., models without covariates) and separate conditional models (i.e., models with covariates). Second, we ran multi-group LGMs with covariates. Third, we estimated joint (bivariate) LGMs and multi-group bivariate LGMs.

#### How do somatic symptoms and family disagreements change over time?

In order to examine how somatic symptoms and family disagreements change over time, we used LGM to fit univariate trajectories for somatic symptoms and family disagreements, respectively. For each univariate trajectory, we fit and compared four unconditional models: (a) an intercept only model (i.e., no growth); (b) a linear growth model; (c) a quadratic growth model; and (d) a piecewise growth model (we included the piecewise growth model to estimate whether there were two stages of growth representing the change from middle to older adulthood). We used the model with the best fit statistics to assess univariate conditional models (i.e., models with covariates). Finally, we dropped non-significant covariates and estimated the final univariate conditional model.

#### Do changes in somatic symptoms and family disagreements differ between depression and community groups?

In order to find out whether changes in somatic symptoms or family disagreements differ between the depression and community groups, we estimated multi-group LGMs to fit unconstrained LGMs and constrained LGMs. The unconstrained LGMs allowed each estimated parameter (i.e., intercepts, slopes, and associations) to be unequal between the depression and community groups. The constrained LGMs set each estimated parameter to be equal across the depression and community groups. Next, for each estimated parameter, we calculated the Satorra-Bentler scaled chi-square difference (i.e., Δχ^2^) [25] between unconstrained LGMs and constrained LGMs. A significant chi-square value indicated that the estimated parameter was significantly different for the depression group compared to the community group. These models were run separately for somatic symptoms and family disagreements.

#### Are changes in somatic symptoms associated with changes in family disagreements over time and does the association differ between depression and community groups?

In order to find out whether changes in somatic symptoms were associated with changes in family disagreements, we used joint (bivariate) LGM with covariates to estimate four associations: (a) the association between the baseline number (i.e., intercept) of somatic symptoms and the baseline number of family disagreements; (b) the association between the baseline number of somatic symptoms and rate of change (i.e., slope) in family disagreements; (c) the association between the baseline number of family disagreements and the rate of change (i.e., slope) in somatic symptoms; and (d) the association between rates of change in the number of somatic symptoms and family disagreements.

In order to examine whether the association between changes in somatic symptoms and family disagreements differed between the depression and community groups, we used multi-group bivariate LGMs to fit unconstrained bivariate LGMs and constrained bivariate LGMs, and then calculated the Satorra-Bentler scaled chi-square difference between unconstrained bivariate LGMs and constrained bivariate LGMs. Again, a significant chi-square value indicated that the estimated association was significantly different between the depression and community groups.

Basic descriptive statistics and correlation analyses were conducted using SAS. All analyses related to LGMs were performed in Mplus 6.21 [26]. Parameter estimation and missing data were handled by maximum likelihood estimation with robust standard errors and missing at random, respectively. A comparative fit index greater than .90 [27], a root mean square error of approximation between .05 and .08 [28], a standardized root mean square residual less than .08 [29], and a smaller Bayesian information criterion (BIC) [30] indicate an adequate model fit. Unstandardized estimates are reported for all LGMs.

## Results

### Descriptive characteristics of depression and community groups and correlations with outcome variables

Table 1 describes participant characteristics at baseline and descriptive statistics for somatic symptoms and family disagreements at each time point. The two groups were not statistically different in age, gender, or race/ethnicity, probably because of the matching sample selection procedure. The depression group reported statistically significant fewer years of education and more medical conditions than the community group, with the effect size (Cohen’s d) being .35 and .49, respectively. The average number of somatic symptoms and family disagreements were consistently greater among the depression than the community group at each time point (except for family disagreements at T5), with the effect size (Cohen’s d) ranging from .19 to 1.11.

**Table 1:** Descriptive characteristics for depression (*N* = 423) and community groups (*N* = 424)

	Depression*M* (*SD*)	Community *M* (*SD*)	t-tests or *χ* ^2^ tests	Cohen’s d	Depression Median (Range)	Community Median (Range)
Covariates						
Age in years	39.9 (14.1)	39.4 (15.6)	−.56^ns^		37 (18–83)	35 (18–88)
Female %	56 %	54 %	.15^ns^			
White %	84.6 %	88.4 %	2.60^ns^			
Education in years	13.3 (2.3)	14.1 (2.3)	4.95^***^	.35	13 (8–17)	14 (8–17)
Medical conditions	.97 (1.22)	.46 (.84)	−7.02^***^	.49	1 (0–8)	0 (0–6)
Somatic symptoms						
T1	5.5 (3.17)	2.3 (2.54)	−16.5^***^	1.11	5 (0–12)	1.5 (0–12)
T2	4.7 (3.27)	2.2 (2.60)	−12.18^***^	.85	4 (0–12)	1 (0–12)
T3	4.1 (3.14)	2.1 (2.39)	−9.61^***^	.72	3 (0–12)	1 (0–11)
T4	3.6 (3.00)	1.7 (2.08)	−9.48^***^	.74	3 (0–12)	1 (0–10)
T5	3.8 (2.91)	2.1 (2.44)	−7.3^***^	.63	3 (0–12)	1 (0–11)
Family disagreements						
T1	3.5 (2.88)	2.6 (2.58)	−4.6^***^	.33	3 (0–14)	2 (0–14)
T2	3.2 (2.75)	2.4 (2.46)	−4.22^***^	.31	3 (0–14)	2 (0–13)
T3	2.9 (2.81)	2.4 (2.46)	−2.66^**^	.19	2 (0–12)	2 (0–10)
T4	2.8 (2.68)	2.1 (2.17)	−3.38^***^	.29	2 (0–14)	2 (0–10)
T5	2.1 (2.40)	2.0 (2.28)	−.6^ns^		1 (0–10)	1 (0–10)

T1 to T5 = baseline, 1 year, 4, 10, and 23 years follow-up

^*^ *p* < .05 ^**^ *p* < .01 ^***^ *p* < .001. ns nosignificant

**Table 2:** Correlation between somatic symptoms and family disagreements

Correlation between Somatic Symptoms and Family Disagreements	Depression Group	Community Group
T1	0.23^***^	0.20^***^
T2	0.17^***^	0.19^***^
T3	0.18^**^	0.26^***^
T4	0.23^***^	0.20^***^
T5	0.08	0.31^***^

Note: ^**^ *p* < .01 ^***^ *p* < .001

The number of somatic symptoms was positively associated with the number of family disagreements at each time point, with correlations ranging from .17 to .31, *p*s < .001 (with the exception of the depression group at T5. See Table 2). Older participants reported significantly fewer family disagreements, but age was not associated with somatic symptoms in either group. Gender was not significantly associated with somatic symptoms or family disagreements and therefore was not included as a covariate in any LGM analyses. In both groups, individuals with more years of education reported fewer somatic symptoms, and those with more medical conditions reported more somatic symptoms.

### How do somatic symptoms change over time and do changes differ between depression and community groups?

The predicted unconditional piecewise LGMs (i.e., without covariates) had the best model fit statistics for both the depression and community groups, suggesting that the trajectory of somatic symptoms followed two stages of growth. The final conditional piecewise LGMs, which included the significant associations of education and medical conditions with baseline number of somatic symptoms, had the best model fit statistics for both groups.

**Fig. 1:**
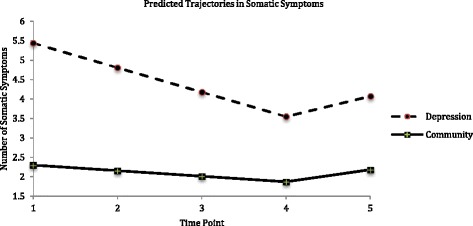
Predicted LGM trajectories in somatic symptoms in depression and community groups. Time Point 1 (T1) to Time Point 5 (T5) = baseline, 1 year, 4, 10, and 23 years follow-up. Intercept = baseline number; slope 1 = rate of change from T1 to T4; slope 2 = rate of change from T4 to T5. Somatic symptoms: depression group (intercept = 5.1, *p* < .001; slope 1 = −.62, *p* < .001; slope 2 = .50, *p* < .01; association between intercept and slope 1 = −.57, *p* < .001); community group (intercept = 2.6, *p* < .001; slope 1 = −.13, *p* < .01; slope 2 = .34, *p* < .01; association between intercept and slope 1 = −.37, *p* < .001)

Figure 1 depicts the predicted conditional piecewise LGMs for somatic symptoms, and lists the statistics for predicted average baseline numbers (i.e., intercepts) and rates of change (i.e., slopes) for both the depression and community groups. The number of somatic symptoms decreased from T1 to T4 (i.e., slope 1), but increased from T4 to T5 (i.e., slope 2). In addition, compared to the community group, the depression group reported more somatic symptoms at each time point.

Results of the multi-group LGMs show that, compared to the community group, the depression group reported a significantly higher baseline number (i.e., intercept) of somatic symptoms, Δχ^2^ (1, *N* = 847) = 87.31, *p* < .001, and had a greater decrease from T1 to T4 (i.e., slope 1), Δχ^2^ (1, *N* = 847) = 44.65, *p* < .001, but did not have a greater increase from T4 to T5 (i.e., slope 2), Δχ^2^(1, *N* = 847) = .72, *p* = .40. Compared to individuals with fewer baseline somatic symptoms, individuals with more baseline levels of somatic symptoms had a *faster* decline in somatic symptoms between T1 and T4 (see Fig. 1 for statistics). This association was similar for both the depression and community groups, Δχ^2^(1, *N* = 847) = .59, *p* = .44.

### How does family disagreements change over time and do changes differ between depression and community groups?

The predicted unconditional linear LGMs for family disagreements had the best model fit statistics for both the depression and community groups, suggesting that the trajectory of family disagreements linearly decreased over time. The final conditional linear LGMs, which included the significant association of age with the intercept of family disagreements, had a better fit than the unconditional LGMs for both groups.

**Fig. 2:**
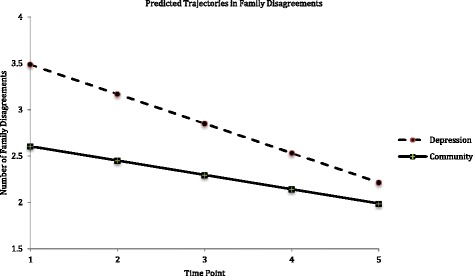
Predicted LGM trajectories in family disagreements in depression and community groups. Time Point 1 (T1) to Time Point 5 (T5) = baseline, 1 year, 4, 10, and 23 years follow-up. Intercept = baseline number; slope = rate of change from T1 to T5. Family disagreements: depression group (intercept = 3.5, *p* < .001; slope = −.35, *p* < .001; association between intercept and slope = −.99, *p* < .001); community group (intercept = 2.6, *p* < .001; slope = −0.16, *p* < .001; association between intercept and slope = −.47, *p* < .001)

Figure 2 depicts the predicted conditional linear LGMs for family disagreements, and lists the statistics for predicted average baseline numbers (i.e., intercepts) and rates of change (i.e., slopes) for both groups. Fig. 2 also shows that, in comparison to the community group, the depression group consistently reported more family disagreements.

Results of the multi-group LGMs suggest that, compared to the community group, the depression group had a significantly higher baseline number (i.e., intercepts) of family disagreements, Δχ^2^(1, *N* = 847) = 25.35, *p* < .001, and had a greater decrease in the number of family disagreements over time (i.e., slope), Δχ^2^ (1, *N* = 847) = 9.66, *p* < .01. For both groups, compared to individuals with fewer baseline numbers of family disagreements, individuals with a higher baseline number of family disagreements had a faster decline in family disagreements over time (see Fig. 2 for statistics). This association was stronger in the depression group, Δχ^2^ (1, *N* = 847) = 6.33, *p* < .05.

### Are changes in somatic symptoms associated with changes in family disagreements over time and do the associations differ between depression and community groups?

**Fig. 3:**
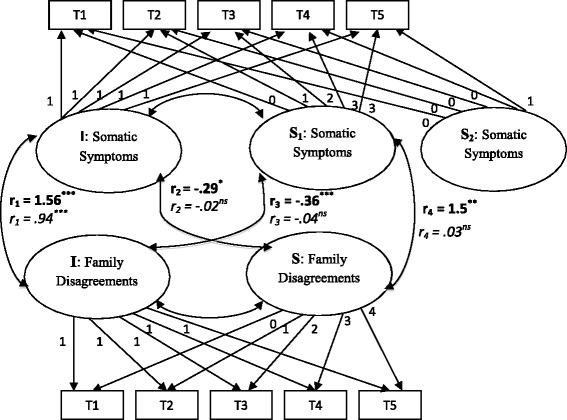
Joint trajectories of somatic symptoms and family disagreements in depression and community groups. **Bold = depression group**, *italic = community group*. Unstandardized coefficients are presented. ^*^ *p* < .05 ^**^ *p* < .01 ^***^ *p* < .001. T1 to T5 = baseline, 1 year, 4, 10, and 23 years follow-up. I: Intercept (i.e., baseline number) (factor loading fixed at 1); S_1_: Slope 1 (i.e., rate of change from T1 to T4) (factor loadings fixed at 0, 1, 2, and 3); S_2_: Slope 2 (i.e., rate of change from T4 to T5) (factor loadings fixed at 0 and 1); S: Slope (i.e., rate of change from T1 to T5) (factor loadings fixed at 0, 1, 2, 3, and 4). *r*_*1*_: association between intercept of somatic symptoms and intercept of family disagreements; *r*_*2*_: association between intercept of somatic symptoms and slope of family disagreements; *r*_*3*_: association between intercept of family disagreement and slope 1 of somatic symptoms; *r*_*4*_: association between slope 1 of somatic symptoms and slope of family disagreements

The bivariate LGMs with covariates had good model fit statistics for both the depression and community groups. Because somatic symptoms followed two separate rates of change over time (slope 1 and slope 2), and because slope 2 only included two time points, the association between the intercept of family disagreements and slope 2 of somatic symptoms was set to zero. Fig. 3 describes the predicted associations between the baseline number and rate of change in somatic symptoms and family disagreements from the bivariate LGMs for both groups. (Covariates were not shown in Fig. 3, but were included in the analyses.)

Overall, findings from the multi-group bivariate LGMs suggest that the association between the number of somatic symptoms and the number of family disagreements was stronger in the depression group than in the community group. Except for the association between the baseline number of somatic symptoms and family disagreements (i.e., *r*_*1*_), all other associations were significantly or marginally stronger in the depression than the community group. Thus, compared with individuals in the community group, individuals in the depression group who had a greater baseline number of somatic symptoms reported a somewhat greater decline in family disagreements over time (i.e., *r*_*2*_), Δχ^2^(1, *N* = 847) = 2.98, *p* < .10. In addition, individuals in the depression group who had a greater baseline number of family disagreements, experienced a greater decline in somatic symptoms (i.e., *r*_*3*_), Δχ^2^(1, *N* = 847) = 3.78, *p* = .05. Finally, for individuals in the depression group, greater reduction in somatic symptoms was significantly associated with a greater reduction in family disagreements (i.e., *r*_*4*_), Δχ^2^(1, *N* = 847) = 4.67, *p* < .05.

## Discussion

To our knowledge, this is the first study to report on longitudinal changes in somatic symptoms and family disagreements among depression and community groups over 23 years. Somatic symptoms changed in a non-linear pattern for both groups, with significant decreases from baseline to 10-year follow-up and significant increases from the 10-year to the 23-year follow-up. The average baseline number and the rates of change in somatic symptoms were both greater in the depression group compared to the community group. Family disagreements decreased over time in both groups, but the depression group had significantly higher baseline levels and greater decreases in family disagreements compared to the community group. The association between changes in somatic symptoms and changes in family disagreements was stronger in the depression than in the community group. The trajectories of somatic symptoms and family disagreements were parallel in the depression group from the baseline to the 10-year follow-up, with a larger decrease in somatic symptoms associated with a larger decrease in family disagreements. However, the trajectories of somatic symptoms and family disagreements were not parallel in the community group.

Somatic symptoms followed a non-linear trajectory over time in both the depression and community groups. There is no obvious explanation for the slight decline in self-reported somatic symptoms between baseline and the 10-year follow-up in the community group. However, for the depression group, the treatment of depression may have contributed to the relatively steep decreasing trajectory. The increase in somatic symptoms from the 10-year to the 23-year follow-up is most likely due to participants making the transition from middle to late adulthood (mean age at the 10-year follow-up: depression vs. community, 47.6 vs. 47.6; mean age at the 23-year follow-up: 57.9 vs. 58.3). Therefore, the increasing trajectory of somatic symptoms could be due to age-related declines in physical health or age-related increases in vulnerabilities for stress-related immunological impairments [31–33].

Existing theories also offer several potential explanations for the findings regarding why participants reported fewer family disagreements over time. For example, the theory of selective optimization with compensation suggests that as individuals age, they try to maximize positive experiences and minimize negative experiences (e.g., family disagreements) [34]. It is also possible that, as hypothesized by accommodation theory [35], family members become more tolerant and circumspect over time, thus making family disagreements less salient and, in turn, less likely to be reported.

In general, findings regarding the interrelationships among somatic symptoms, family disagreements, and depression are also supported by past research. In the present study, the depression group reported a greater baseline number and a greater rate of change in both somatic symptoms and family disagreements. The association between changes in somatic symptoms and changes in family disagreements was also stronger in the depression group. These findings are consistent with previous studies indicating that depression is positively associated with marital distress [36], interpersonal conflict [37], and somatic symptoms [18, 38]. However, it is possible that the individuals in the depression group showed a greater decrease in the number of somatic symptoms and family disagreements because they received treatment for depression [38–40]. In addition, there is the possibility that because the depression group had higher baseline levels of somatic symptoms and family disagreements, they simply had more opportunity for improvement than the community group.

As with all research, this study has some limitations. For example, family disagreements were only measured from the point of view of one family member. Future studies should assess family disagreements from multiple perspectives. Unlike patients’ self-report for somatic symptoms, physicians’ diagnoses mostly capture severe somatic symptoms. Therefore, results of the present study might not be generalizable to those patients whose somatic symptoms were diagnosed by physicians. Some of the variables in our analyses may not have been normally distributed, which violates one of the assumptions of this modeling approach. Given that depression is often comorbid with other psychiatric disorders [41], it is also possible that other psychiatric conditions (e.g., anxiety) are responsible for our results. In addition, given that the level of depression is determined by the number and severity of a variety of symptoms, it is possible that our measure of depression overlaps somewhat with our measure of somatic symptoms. The analyses do not allow for inferences about causal relationships or reciprocal effects between somatic symptoms and family disagreements. In future studies, the use of a more dynamic structural model may help unravel the causal interrelationships between somatic symptoms and family disagreements over time. It will also be very interesting for future studies to examine the association between the emergence of new somatic symptoms/family disagreements (or the discontinuation of previous ones) and corresponding changes in the other dimension. Such an examination would provide valuable information on the causal relationships between specific somatic symptoms and subsequent disagreements and vice versa. Also, it would be of interest for future work to examine the associations between changes in specific medical conditions over time and family life quality. Despite these limitations, we believe this study contributes to the existing literature by being the first to examine the longitudinal relationship between various forms of somatic symptoms and family disagreements over 23 years in both depression and community groups.

## Conclusion

In summary, our findings suggest that somatic symptoms and family disagreements are inter-related and change together over time. Therefore, it may be possible to alleviate somatic symptoms by resolving family disagreements or to reduce family disagreements by alleviating somatic symptoms through appropriate interventions. Given the stronger association between somatic symptoms and family disagreements in the depression group, such interventions may be especially useful for individuals with depression. Ideally, these interventions would be implemented in primary care settings where most individuals with physical symptoms and mental health problems seek treatment.

## Abbreviations

LGM: Latent growth curve modeling
